# Analysis of the Characteristics and Evolution Modes of PM_2.5_ Pollution Episodes in Beijing, China During 2013

**DOI:** 10.3390/ijerph120201099

**Published:** 2015-01-22

**Authors:** Ci Song, Tao Pei, Ling Yao

**Affiliations:** State Key Laboratory of Resources and Environmental Information System, Institute of Geographic Sciences and Natural Resources Research, Chinese Academy of Sciences, Beijing 100101, China; E-Mails: songc@lreis.ac.cn (C.S.); peit@igsnrr.ac.cn (T.P.)

**Keywords:** PM_2.5_ concentration, PM_2.5_ pollution episodes, seasonal variations, peak patterns, air quality

## Abstract

Fine particulate matter (PM_2.5_) has been recognized as a serious hazard linked to deleterious health effects. In this study, all PM_2.5_ Pollution Episodes (PPEs) in Beijing during 2013 were investigated with hourly PM_2.5_ observations from the Olympic Sport Center site, and then their characteristics and evolution modes analysed. Results show that 80 PPEs, covering 209 days, occurred in Beijing during 2013. Average PM_2.5_ concentrations during PPEs were almost twice (1.86) the annual mean value, although the PPEs showed significant seasonal variations. The most hazardous PPEs tended to occur in winter, whereas PPEs with long duration occurred in autumn. The PPEs could be divided into six clusters based on their compositions of different pollution levels, which were strongly related to meteorological factors. We used series peaks of PM_2.5_ concentrations to analyse the evolution modes of PPEs and found that the more peaks there were within the evolution mode, the longer the duration, and the higher the average and maximum PM_2.5_ concentrations. Each peak within a PPE can be identified by “rise” and “fall” patterns. The “rise” patterns are widely related to relative humidity, whereas the “fall” patterns are affected principally by wind speed for one-peak PPEs and boundary layer height for multi-peak PPEs. The peak patterns cannot be explained fully by meteorological factors; however, they might also be closely related to complex and diversified human activities.

## 1. Introduction

The combination of urbanisation, industrialisation, and population growth in China has led to a remarkable increase in emissions, and the problem of air pollution has received increasing attention because of its influence on daily life via the climate, environment, visibility, and health. 

One of the most harmful air pollutants is particulate matter (PM). Inhalable particles (PM_10_) can penetrate deep inside the lung, which not only decreases the function of the respiratory and cardiovascular systems, but also increases mortality from pollution-related disease; however, PM_2.5_ is associated more with adverse health effects than the coarser particles are [1,2,3]. An increasing number of studies have been focused on the variation of PM_2.5_ concentration. Some studies have considered the chemical composition of PM_2.5_, including elemental constituents, water-soluble ions, and organic carbon [4,5,6,7]. Other research has attempted to describe the spatiotemporal distributions of PM from site monitoring data, including spatial patterns [8,9,10], diurnal variations [11], and annual periods and trends [12,13,14] of PM concentrations, and to demonstrate their relationships with confounding meteorological factors [15,16]. 

Beijing, the capital of China, is one of the cities in the world most seriously affected by the problem of air pollution, and considerably higher PM_2.5_ concentrations have been observed there. For example, a value of 101 μg/m^3^ was found in 2000 in the study by Zheng *et al.* [17], which is similar to the value of 115–127 μg/m^3^ observed from 1999 to 2000 in the study by He *et al.* [4] These values are much higher than the 12 μg/m^3^ found in the United States from 2000 to 2007 [18], and the value of 8–25 μg/m^3^ observed regionally in Switzerland in 1998–2001 [19]. Wang *et al.* [20] and Zheng *et al.* [17] have demonstrated that the major sources of PM_2.5_ in Beijing are coal combustion, traffic exhaust, dust, and industrial activities. In Beijing, research has increasingly been undertaken on the seasonal or diurnal changes [21] of PM_2.5_ concentrations series and their emission sources [22,23]. Furthermore, other investigations have demonstrated the impact of meteorological factors on PM_2.5_ concentrations [24,25], and the seriously damaging effect PM_2.5_ pollution can have on health in Beijing [26]. However, few studies have focused on the evolution process of each specific PM_2.5_ Pollution Episode (PPE). These evolution processes, evolving through several different stages, such as emergence, stability, and dispersion, provide a comprehensive depiction of each PPE. These evolution modes can be used to retrieve historical PPE records and predict future PPEs. Thus, in this research, we use PM_2.5_ observations to further our understanding of PPEs.

For this study, hourly PM_2.5_ observations were collected continuously at an urban site in Beijing for 13 months (1 February 2013–28 February 2014). In this paper, we provide the definition of PPEs and analyse the characteristics of each PPE with these records. Furthermore, we extract the evolution mode of each PPE, and explore its relationship with confounding meteorological factors. To identify the evolution mode of each PPE, we generalise the PM_2.5_ concentration series in each PPE using a Perceptually Important Points (PIPs) extraction method, and classify all the PPEs into one of five categories according to its evolution modes. Each category is analysed to compare the influence of variations of the meteorological factors. 

The remainder of the paper is organised as follows: the data and methods are described in Section 2. After the basic characteristics of PPEs are defined in Section 3.1, we divide the PPEs into different clusters based on their different compositions in Section 3.2. In Section 3.3, we identify the evolution mode of each PPE and analyse its relationships with meteorological factors. Discussions are also presented in these sections. Finally, conclusions are drawn in Section 4.

## 2. Data and Method

### 2.1. Data Source

Two kinds of data sources were used in this study, the details of which, together with an appraisal of their uncertainties, are described in the following.

#### 2.1.1 Ground Observations 

We collected hourly PM_2.5_ concentration observations at the Olympic Sport Centre site (39.982 °N, 116.397 °E) from continuous particulate monitor (BAM-1020, Supplementary Information, Table S2) during the period from 1 February, 2013 to 28 February, 2014, which provided a data set of 6650 records. There were 2782 missing hourly values, which included 46 entire days. Where possible, parts of the records, with less than 6 h missing, were recovered using linear interpolation. Entire days of missing data were not used in the following analysis.

#### 2.1.2 Meteorological Data 

ERA-Interim is the global atmospheric reanalysis data produced by the European Centre for Medium-Range Weather Forecasts [27]. It extends back to 1979, and the analysis continues to be extended forward in near-real time. A more detailed description of the ERA-Interim product archive can be found in the paper by Berrisford *et al.* [28]. Simmons *et al.* [29] have found that ERA-Interim data agree well with the Climatic Research Unit and Hadley Centre analyses of monthly station temperature data (CRUTEM3), and the correlations between the CRUTEM3 and ERA-Interim data in North America and Asia exceed 99% [14]. In our study, gridded observation records at 3-hourly intervals including wind speed (WS), dew point temperature (DP), surface temperature (ST), and boundary layer height (BLH) were used. The relative humidity (RH) was calculated using the Goff–Grattch equation with the DP and ST records.

### 2.2. Definition of PM_2.5_ Pollution Episodes (PPEs)

In this study, each PPE is defined with a start hour and an end hour in the PM_2.5_ concentration series. The start hour of a PPE is defined as the first span of at least 12 h with PM_2.5_ concentration > 75 μg/m^3^, and the end hour of the PPE is defined as the first span of 6 h after the start hour with PM_2.5_ concentration < 75 μg/m^3^. In our definition, the duration of each PPE is at least 12 h.

**Figure 1:**
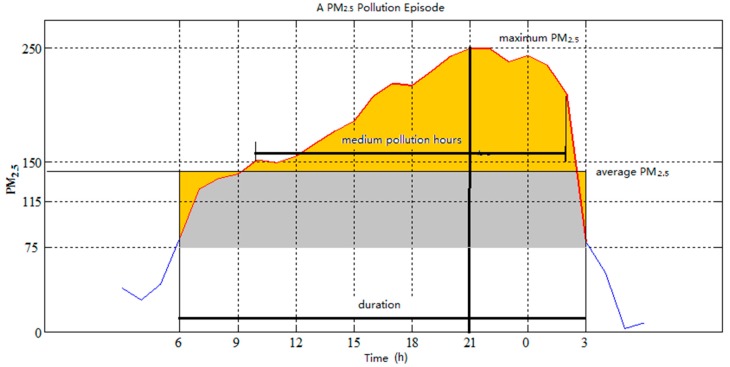
Definitions of PM_2.5_ Pollution Episode.

Six indices were employed in this study to analyse the basic characteristics of each PPE, the details of which are given in Table 1.

**Table 1:** Definitions of six indices of PM_2.5_ Pollution Events.

Index	Description	Definition	Unit
Duration	Length of hours in a PPE	$I_{e}-I_{b}$	h
Ave_PM_2.5_	average PM2.5 concentration in a PPE	$\sum_{t=T_{b}}^{T_{e}}C_{t}/(T_{e}-T_{b})$	*µg*/*m*^3^
Max_PM_2.5_	Maximum PM_2.5_ concentration in a PPE	$max(C_{t}),t\in [I_{b},I_{e}]$	*µg*/*m*^3^
Light-polluted cumulative hours	Cumulative hours with PM_2.5_ concentration less than 115 μg/m^3^ in a PPE ^a^	$\left|\{t|C_{t}<115\mu g\}\right|$	h
Medium-polluted cumulative hours	Cumulative hours with PM_2.5_ concentration between 115 μg/m^3^ and 150 μg/m^3^ in a PPE ^a^	$\left|\{t|115\mu g\leq C_{t}<150\mu g\}\right|$	h
Heavy-polluted cumulative hours	Cumulative hours with PM_2.5_ concentration more than 150 μg/m^3^ in a PPE ^a^	$\left|\{t|150\mu g\leq C_{t}\}\right|$	h

^a ^The PPE polluted hours of different class are defined according to Ambient air quality standards [30]

Here, *T_b_* and *T_e _*denote the start and end hours of a PPE, respectively, and *C_t_* represents the PM_2.5_ concentration record in time *t*. Basic statistics regarding these indices have been analysed, and different combinations of these indices used to describe different characteristics of PPEs, including health hazard levels and compositions. Simple classification and Time Series Clustering methods have been used in this study.

### 2.3. Identification of Evolution Mode for Each Pollution Episode (PPE)

To determine the evolution mode of the PPEs, the PIPs of each PPE were identified. The concept of PIPs describes the general shape of the time series, when a data point that has greater domination over the overall shape of the series is considered more important. For a given PPE, which is represented by the PM_2.5_ concentration sequence *P*, the first two PIPs will be the first and last points of *P*. The next PIP will be the point in *P* with maximum distance to the first two PIPs. The fourth PIP will then be the point in *P* with maximum distance to its two adjacent PIPs, either in between the first and second PIPs or in between the second and last PIPs. This process of locating the PIPs continues until all the points in *P* are attached to a list (Supplementary Information Figure S1a). Here, we used Euclidean Distance (PIP-ED, [31]) to evaluate the importance of the PIPs in each PPE (Supplementary Information Figure S1b). Points with PIP-ED larger than a threshold have been preserved.

## 3. Results and Discussion

### 3.1. Basic Characteristics of PM_2.5_ Pollution Episodes (PPEs)

We identified 80 PPEs covering 209 days in Beijing during the study period using the method mentioned in 2.2, and these PPEs occupied 45% of the hours of the entire year. Most PPEs occur in January, February, and September (Supplementary Information Figure S2). There are 27 PPEs with an average PM_2.5_ concentration < 115 μg/m^3^, 22 PPEs with an average PM_2.5_ concentration of 115–150 μg/m^3^, and 31 hazardous PPEs with an average PM_2.5_ concentration > 150 μg/m^3^ observed during this period. Average PM_2.5_ concentrations during PPEs are almost twice (1.86) the annual mean value of the entire year (about 87.81 ± 68.43 μg/m^3^, Supplementary Information, Table S1, Figure S3) compared with other mega cities in China, such as Shanghai (103.07 μg/m^3^ in Baoshan and 62.25 μg/m^3^ in the Putuo district) [10] and Nanjing (114.88 μg/m^3^) [32].

(Supplementary Information, Figure S4) shows the PPEs with average PM_2.5_ concentrations of different levels in each season. PPEs are frequent in winter, but relatively fewer in summer. Light PPEs are widely observed in each season, while medium PPEs are more likely in summer and winter. Moreover, over half the hazardous PPEs occurred in winter (Table 2). 

**Table 2:** Number of PPEs with average PM_2.5_ concentration of different levels in each season.

Season	Light	Medium	Hazardous	*Sum*.
Spring	7	3	5	15
Summer	7	8	5	20
Autumn	8	3	7	18
Winter	5	8	14	27
Cum.	27	22	21	80

The duration of each PPE is displayed in (Supplementary Information, Figure S5). It can be seen that 27 PPEs last for less than 1 day and 33 last for 1 or 2 days. There are 20 PPEs with durations longer than 2 days (Table 3). Supplementary Information Figure S6 shows PPEs with durations of different levels in each season. Most short PPEs appear in spring, medium PPEs are observed in each season (especially summer and winter), and long PPEs are more likely in autumn and less likely in winter.

**Table 3:** Number of PPEs with durations of different levels in each season.

Season	Short	Middle	Long	*Sum*.
Spring	5	6	4	15
Summer	5	9	6	20
Autumn	5	7	6	18
Winter	12	11	4	27
Cum.	27	33	20	80

### 3.2. Clustering of *PM_2.5_ Pollution Episodes* Based on Different Compositions of PM_2.5_ Concentrations

**Figure 2:**
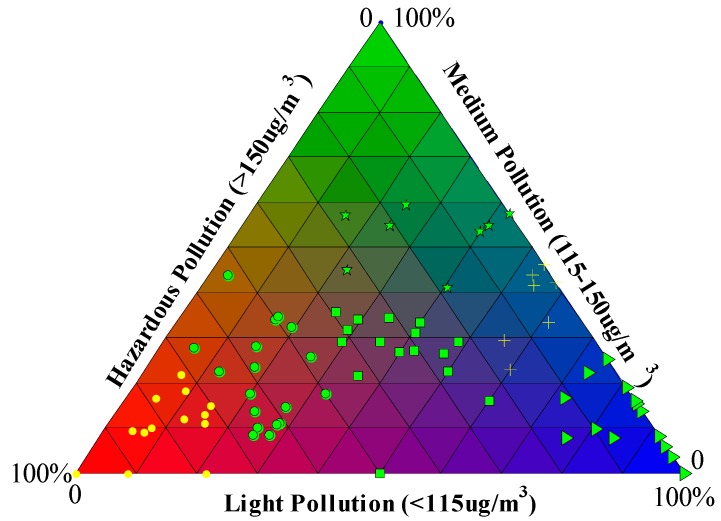
PPE clusters with different compositions of pollution level.

We calculated the duration ratio of different level concentrations for each PPE. According to these ratios, we divided the 80 PPEs into six clusters. Figure 2 shows the different compositions of each PPE and their clusters. Characteristic of these clusters are listed in Table 4. PPEs in the first cluster are represented as yellow dots and these PPEs occur mainly in red triangles, meaning hazardous pollution accounted for a large proportion of the durations. PPEs represented by circles are within the second cluster where over half the durations of the PPEs involved hazardous pollution. Squares in the middle triangles represent the third cluster in which a tripartite situation between the three levels of pollution occurs. Triangles in the fourth cluster mean PPEs with a large ratio of light pollution. The crosses mean that 60% of the duration was light pollution and 30% medium pollution. The pentagrams represent the cluster for which over half the duration involved medium pollution.

Table 5 shows the number of PPEs of different composition clusters in each season. PPEs with a large ratio of hazardous or light pollution (clusters 1 and 4) occur mainly in winter. These phenomena relate mainly to stable weather conditions when RH and BLH have fewer fluctuations [15]. However, PPEs for which about half the duration is medium (cluster 6) or light pollution (cluster 5) appear mainly in summer and autumn. PPEs in cluster 3 with equal durations of each pollution level are mainly distributed in spring and winter. PPEs of cluster 2 with about half the duration involving hazardous pollutions occur less often, which is thought to be related to sudden changes of PM_2.5_ concentrations (Supplementary Information, Figure S7). 

**Table 4:** Characteristic of each PM_2.5_ Pollution Episode in different clusters.

Class	Symbol	Characteristic
C1	Dot	Large ratio of Hazardous Pollution
C2	Circle	50% Hazardous pollution
C3	Square	Three types of pollution with same share
C4	Triangle	Large ratio of Light Pollution
C5	Cross	60% light pollution,30% Medium pollution
C6	Pentagram	50%–60% medium pollution

**Table 5:** Number of PM_2.5_ Pollution Episode from different clusters in each season.

Class	Spring	Summer	Autumn	Winter
C1	4	3	4	7
C2	1	3	2	1
C3	3	2	0	3
C4	1	2	4	6
C5	5	3	6	4
C6	1	7	2	6
Sum	15	20	18	27

### 3.3. Evolution Mode of PM_2.5_ Pollution Events (PPEs)

The evolution mode of PPEs reflects the dynamical variations of PM_2.5_ concentration. One of the most significant characteristics is the appearance of peaks that reflect the accumulation and dispersion processes of PM_2.5 _pollution. 

In our study, we define each peak as a “rise-fall” pattern from PIPs, in which the concentration difference between the peak and valley points should be larger than a threshold. Accordingly, we classified the PPEs into five categories based on the identification of peaks. Supplementary Information Figure S8 displays the characteristics of the evolution modes (red lines) of the PPEs in each category. It can be seen that the more peaks in the evolution mode, the longer the duration, and the higher the average and maximum PM_2.5 _concentrations of the PPEs. These results show clearly the relationships between evolution modes and pollution severity of the PPEs. Table 6 presents the characteristics of the evolution modes of PPEs in each category. The first category has six PPEs with relatively flat fluctuations of the PM_2.5_ concentration series, for which no peak pattern could be identified. These PPEs occur seldom in winter, have short average durations of 18.5 h, and average concentrations of 92 μg/m^3^. The second category displays one-peak patterns for the different PPEs. The average duration in this category is about 27 h, and the average PM_2.5_ concentration is 143.3 μg/m^3^. Most of these PPEs happen in winter with the peaks occurring at night. This is attributed mainly to the higher RH and lower BLH at night [33,34]. Double-peak patterns are evident in the third category with an average duration of over 30 h and average concentration of 145 μg/m^3^. PPEs in this evolution mode are often observed in summer or winter. The fourth and fifth categories show triple-peak patterns and multi-peak patterns, respectively. The durations and average PM_2.5_ concentrations of these two categories are 62 and 84.1 h, and 167.1 and 185 μg/m^3^, respectively.

**Table 6:** Characteristics of evolution modes of PPEs in each category.

Category	Peak Number	PPEs Number	Average Duration	Average Concentration	Maximum Concentration
1	0	6	18.5	91.99	110.67
2	1	38	27	143.3	209.84
3	2	16	35.8	145	263.3
4	3	12	62	167.1	276.6
5	≥4	8	84.1	185	324.5

To establish the relationships between the evolution process of PPEs and meteorological factors, we analyse the correlations between PM_2.5_ concentrations of PPEs in each categories and meteorological factors. In PPEs of the first category (no peak), synchronous observations of RH show a weak correlation with PM_2.5_ concentrations. Although low WS (2.17 m/s) and high average RH (0.72) are favourable meteorological conditions for atmospheric condensation, PM_2.5_ concentrations may hardly rise up to a certain extent in the circumstance of high BLH (529 m). PPEs of the second category are thought to be affected by meteorological factors in three different ways. The first cluster of one peak pattern may be primarily subject to subsidence inversion effect which commonly acts on accumulation process of PM_2.5_ under low BLH weather condition in winter. The second cluster show positive correlations between RH and PM_2.5_ concentrations and negative correlations between WS and PM_2.5_ concentrations and between BLH and PM_2.5_ concentrations. The third cluster presents some exceptions that PM_2.5_ concentrations are positively correlated with WS (Supplementary Information, Table S3b). This indicates that WS is not always blowing off. Sometimes, pollutant emission from the surrounding factories could be blown into downtown area in Beijing. Most of Double-peak PPEs in third category may be sensitive to meteorological factors, when the correlations tend to be higher and the variations are accorded with changes of PM_2.5_ concentrations (e.g., Supplementary Information Figure S9b). Other double-peak PPEs display “small-big peaks” pattern. These PPEs, which show weak correlations between PM_2.5_ concentrations and meteorological factors, are very likely related to a new source of emission or to enhanced continuous emission (Supplementary Information Table S3c). Considering PPE 3 as an example (8 February 2013), the latter significant higher peak is mainly attributed to firecrackers on New Year’s Eve. For the triple-peak and multi-peak PPEs in the fourth and fifth category, the meteorological conditions are relatively stable for atmospheric condensation process when average BLH stay on a lower level and WS is always small. Diurnal cycles of PM_2.5_ concentrations variations could be observed with synchronous daily variability of RH and BLH. During these PPEs, most PM_2.5_ concentrations rise to peak at midnight and fall valley at noon (Supplementary Information Table S3d,e). However, RH in the multi-peak PPEs does not show positive correlations with PM_2.5_ concentrations. That’s may be attributed to the lag effect of atmospheric condensation process. 

For deep studies about the specific evolution process of accumulation and dispersion, we also identified each “rise” and “fall” period in all peaks and compared them with the meteorological factors during the same time. Table 7 shows the correlations between the rate of change of PM_2.5_ concentrations and meteorological factors (WS, RH, and BLH). We can see that RH affects the accumulation process of all categories PPEs except PPEs in the double-peak mode, especially for PPEs with long duration. Negative correlations can be seen between average RH and the rate of rise of PM_2.5_ concentrations. This result is intuitively different from previous studies [15,25], which have demonstrated that pollution accumulates more easily under conditions of higher RH. However, BLH and WS are also important factors affecting the rise pattern of the one-peak and triple-peak processes, respectively. These results are consistent with previous studies [15]. For the dispersion process, a clear negative correlation can be observed between the fall rate of PM_2.5_ and WS in the one-peak pattern because of the “blowing-off” effect. Weak correlations between the rates of change for the double-peak process and meteorological factors confirm the reason as being related to emission source. Furthermore, the dispersion process of the multi-peak process is also highly correlated with RH and BLH. 

**Table 7:** Correlations between weather factors and rise/fall pattern in each category of evolution process.

Correlation	One peak		Double-Peak		Triple-Peak		Multi-Peak	
	Rise	Fall	Rise	Fall	Rise	Fall	Rise	Fall
ave_WS	−0.13	−0.35 ^*^	0.22	−0.05	−0.10	−0.16	0.17	-0.22
ave_RH	−0.42 ^*^	0.06	−0.04	0.10	−0.44 ^*^	0.19	−0.41 ^*^	0.62 ^*^
ave_BLH	−0.23	−0.24	0.19	0.04	0.22	−0.03	0.19	−0.62 ^*^
max_WS	−0.11	−0.19	−0.17	0.08	−0.33 ^*^	−0.02	0.11	−0.08
max_RH	−0.51 ^*^	0.22	−0.14	0.23	−0.57 ^*^	0.24	−0.50 ^*^	0.71 ^*^
max_BLH	−0.34 ^*^	0.03	0.05	0.08	−0.18	0.12	0.10	−0.34 ^*^

^*^ Significance at 0.1 level.

### 3.4. Illustrative Cases

(1) Single peak, wind blowing-off 

A one peak PPE was observed at the end of February, when PM_2.5_ concentrations increased to a hazardous value—441 μg/m^3^ under a suitable weather condition of RH and BLH before 11:00 A.M. However, when WS increased to 8 m/s, PM_2.5_ concentrations had been decreased significantly to a moderate level in 3 h (Supplementary Information Figure S9a). This blowing-off effect should be common in Beijing during winter.

(2) Double peaks, synchronous variations

The double-peak PPE in mid-July show some synchronous variations of PM_2.5_ concentration and meteorological factors. High correlation between these indices can be observed and the peak times of PM_2.5_ are almost accordance with the other three peak (or valley) times (Supplementary Information Figure S9b). This evolution mode of PPE need relatively stable weather conditions with lower WS and higher RH.

(3) Small-Big peak, multi-source emission 

PPE in the Spring Festival show a typical small-big peak pattern. Significant increase of PM_2.5_ concentrations on New Year’s Eve can be observed after midnight (Supplementary Information Figure S9c). RH is suitable for condensation process when pollution emission from firecrackers are enormous. This pattern of PPE always can be seen in Beijing.

## 4. Conclusions

This article documents the characteristics of PM_2.5_ Pollution Episodes (PPEs) and extracts their evolution mode using hourly PM_2.5_ observations obtained in Beijing between 1 February 2013 and 28 February 2014. With the aid of a set of descriptive indices, better understanding of PPEs is gained, and the core conclusions drawn are as follows.

(1) In Beijing, 80 PPEs covering 209 days were identified, 40% of which were hazardous pollution events with an average PM_2.5_ concentration > 150 μg/m^3^; 20 PPEs persisted longer than 2 days. These PPEs show significant seasonal variations with the most hazardous PPEs in winter and longest-lasting PPEs in autumn. Average PM_2.5_ concentrations during the PPEs are almost twice (1.86) the annual mean value of the entire year.
(2) Six clusters of PPEs were established based on their compositions of PM_2.5_ concentrations. PPEs with a large ratio of hazardous or light pollution (clusters 1 and 4) occurred mainly in winter, whereas PPEs for which about half the duration was medium (cluster 6) or light pollution (cluster 5) occurred mainly in summer and autumn. The PPEs that had equal durations of all three pollution levels occurred mainly in spring and winter. These compositions are affected mainly by meteorological factors.
(3) The evolution modes of the PPEs were identified based on the peak patterns that reflect the accumulation and dispersion processes of PM_2.5_ pollution. The greater the number of peaks in the evolution mode, the longer the duration, and the higher the average and maximum PM_2.5_ concentrations PPEs. Each peak in PPE is identified by “rise” and “fall” patterns that reflect the accumulation and dispersion processes of the PPEs, respectively. The rise patterns in each peak are related to RH. The fall patterns in the one-peak PPEs are affected mainly by WS, whereas those in the multi-peak PPEs are related to BLH.

These results suggest that the peak patterns cannot be fully explained by meteorological factors alone, but that they might also be closely related to complex and diverse human activities. Most importantly, these findings are helpful for furthering our understanding of PM_2.5 _pollution mechanisms, and they can be used to improve the accuracy of model simulations of air quality.

## Supplementary Files

Supplementary File 1
