# Supplementary material for: Analysis of the Characteristics and Evolution Modes of PM2.5 Pollution Episodes in Beijing, China During 2013
Source: Int J Environ Res Public Health. 2015 Jan 22;12(2):1099–111. doi: 10.3390/ijerph120201099 (PMC4344657; doi:10.3390/ijerph120201099)
Supplement: Supplementary File 1 [file ijerph-12-01099-s001.pdf]

Analysis of the Characteristics and Evolution Modes of $PM_{2.5}$ Pollution Episodes in Beijing, China during 2013

1. Data

Table S1. Mean, median and 95% confidence intervals of $PM_{2.5}$ concentrations in annual and different seasons.

$PM_{2.5}$ Concentration	Mean (μg/m^3)	Median (μg/m^3)	95% Confidence Intervals
Annual	87.81	68.4	80.58–95.03
Spring	84.37	67.11	70.01–98.72
Summer	79.86	65.22	68.78–90.94
Autumn	80.02	59.21	65.51–94.54
Winter	98.91	80.23	82.01–115.81

Table S2. Specifications of BAM-1020.

Parameter	Specification
Operating Principle	Measures ambient particulate concentrations using beta ray attenuation
Performance	
Accuracy	Exceeds US-EPA Class III $PM_{2.5}$ FEM standards for additive and multiplicative bias
Measurement Resolution	0.1 μg/m^3
Display Resolution	1 μg/m^3
Lower Detection Limit (2) 1 h	<4.8 μg/m^3 (less than 4.0 μg/m^3 typical)
Lower Detection Limit (2) 24 h	<1.0 μg/m^3
Standard Range	0–1.000 mg/m^3 (0–1000 μg/m^3)

2. Method for Identification of Evolution Mode

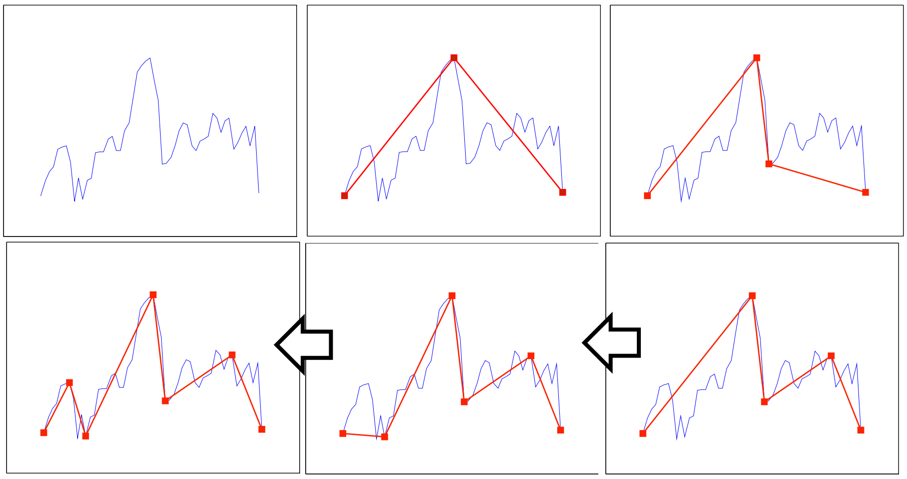

(a)

Figure S1. *Cont.*

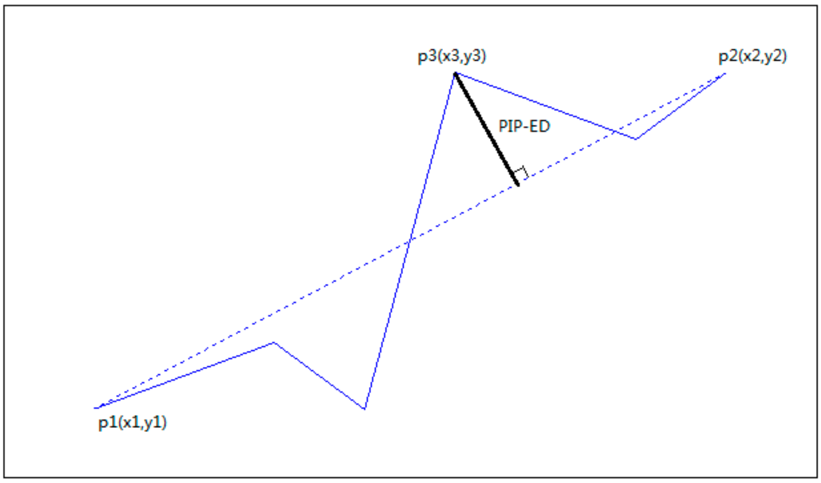

(**b**)

Figure S1. (**a**) Process of perceptually important points extraction; (**b**) perceptually important points-euclidean distance.

3. Characteristics of $PM_{2.5}$ Pollution Episodes

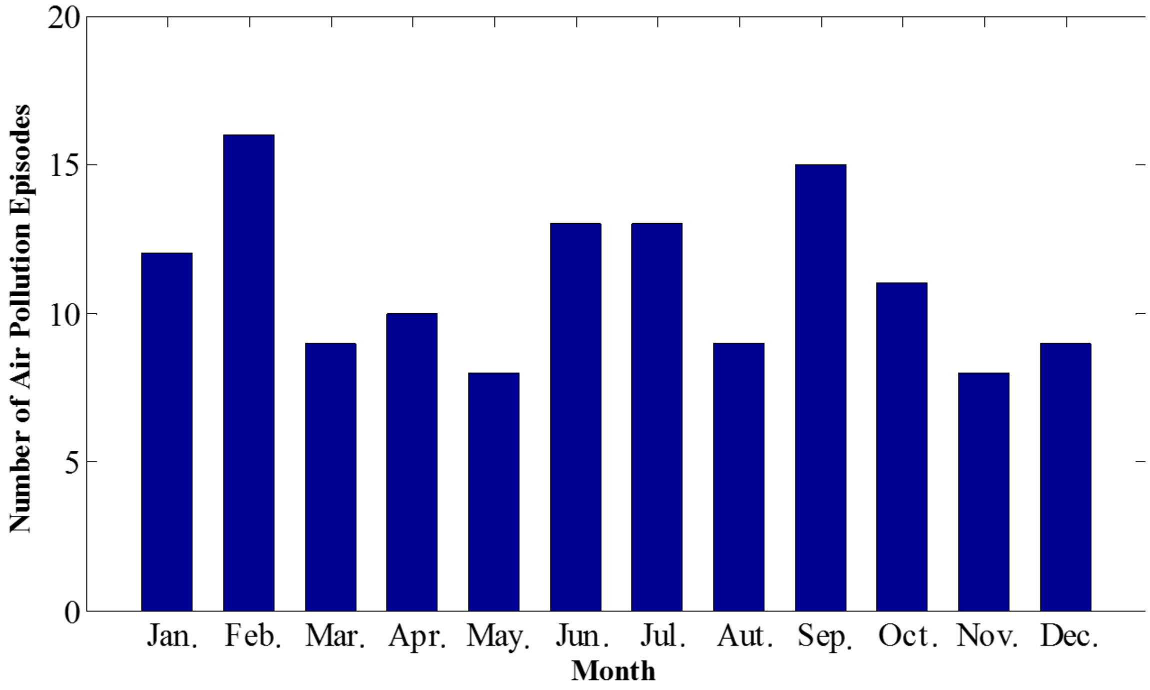

Figure S2. Number of PM2.5 Pollution Episodes (PPEs) in each month.

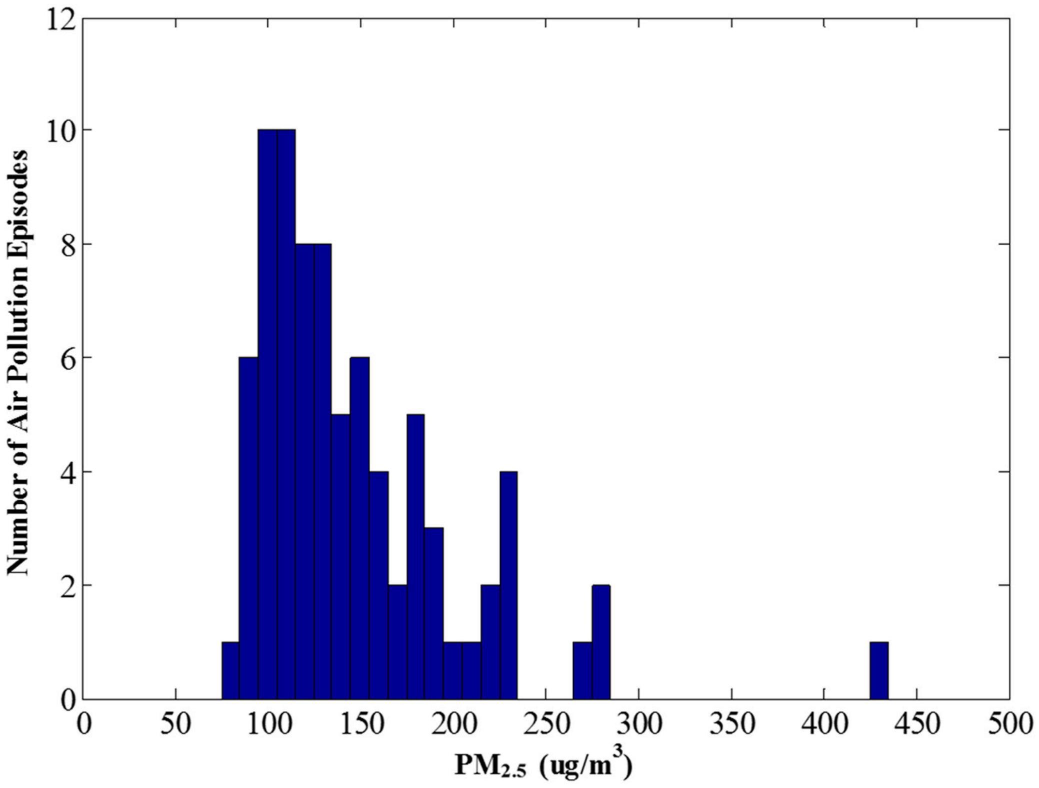

Figure S3. Average $PM_{2.5}$ concentrations of $PM_{2.5}$ pollution episodes.

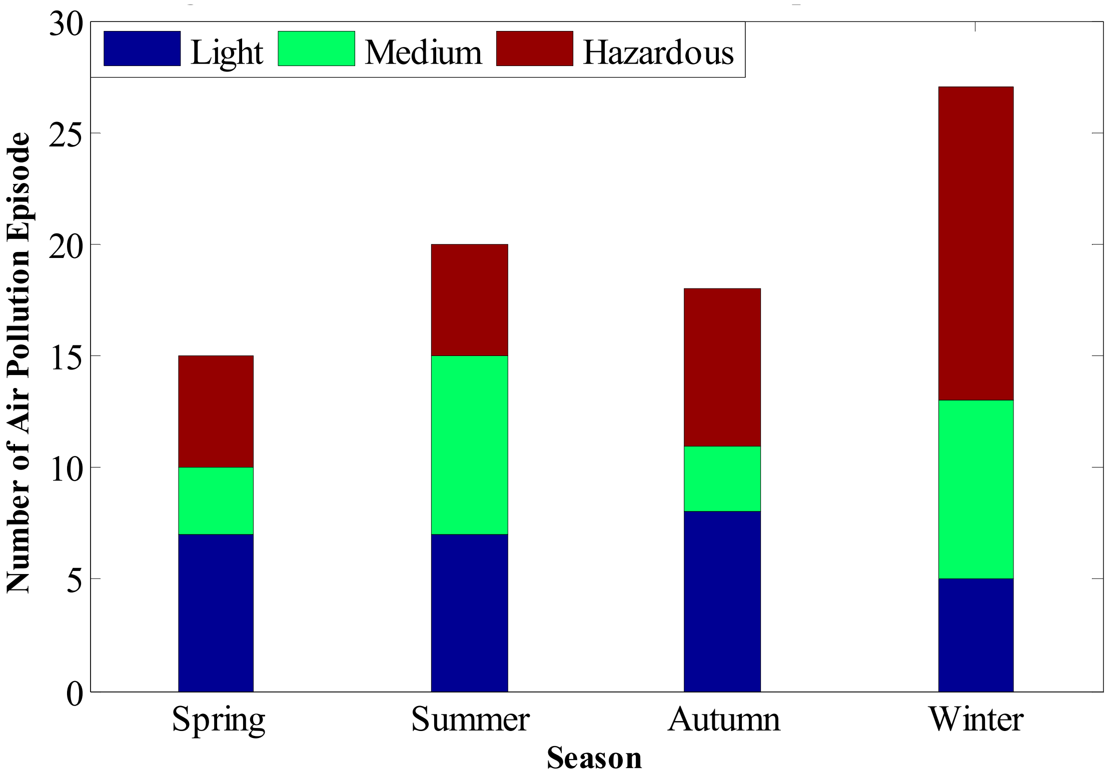

Figure S4. $PM_{2.5}$ Pollution Episodes with average $PM_{2.5}$ concentrations of different levels in each season.

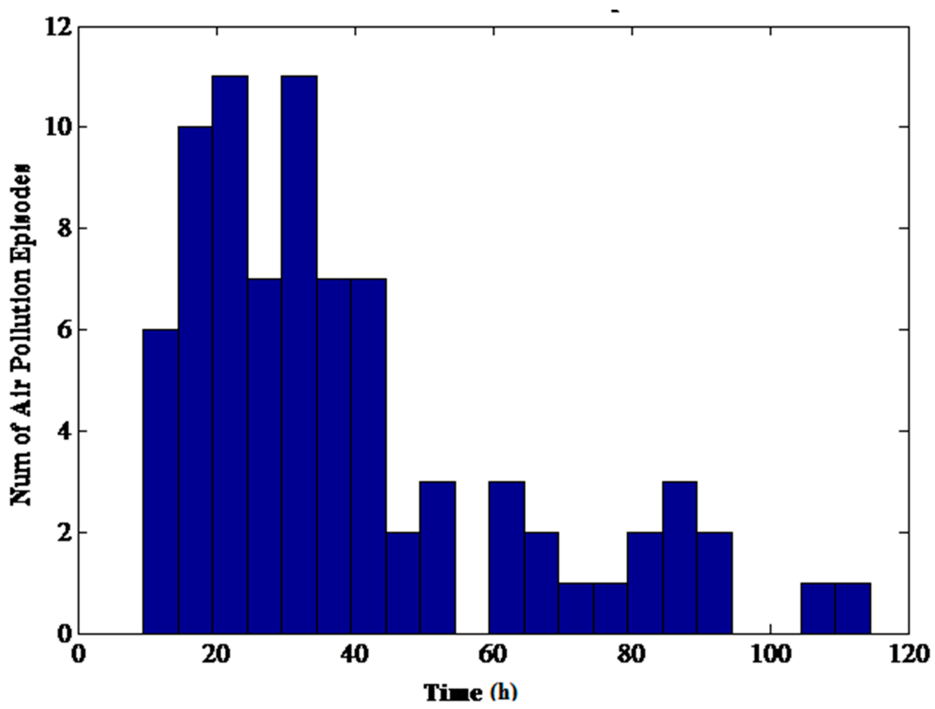

Figure S5. Durations of $PM_{2.5}$ pollution episodes.

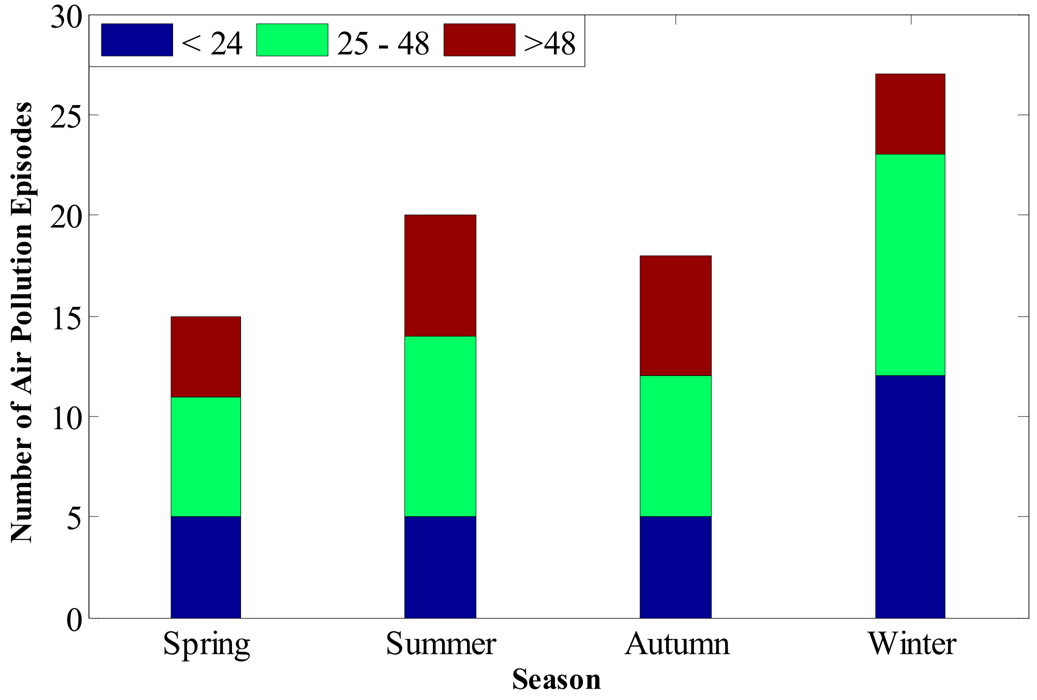

Figure S6. $PM_{2.5}$ Pollution Episodes with durations of different levels in each season.

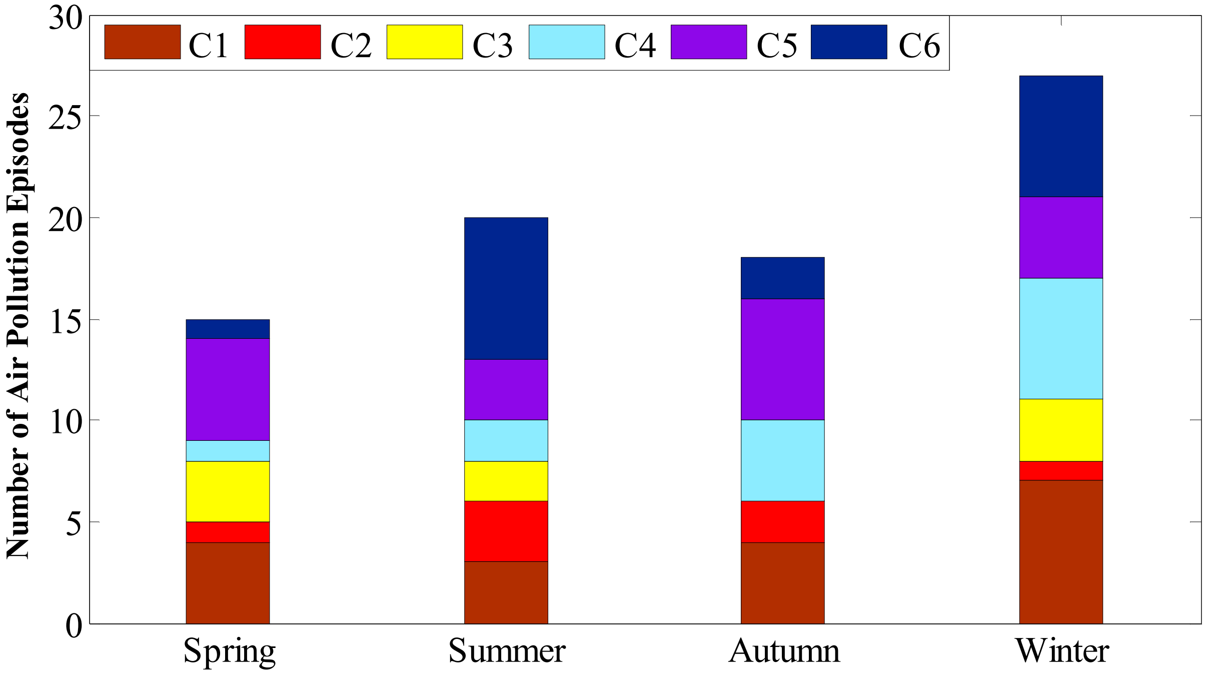

Figure S7. Number of $PM_{2.5}$ Pollution Episodes from different clusters in each season.

4. Evolution Mode of $PM_{2.5}$ Pollution Events (PPEs)

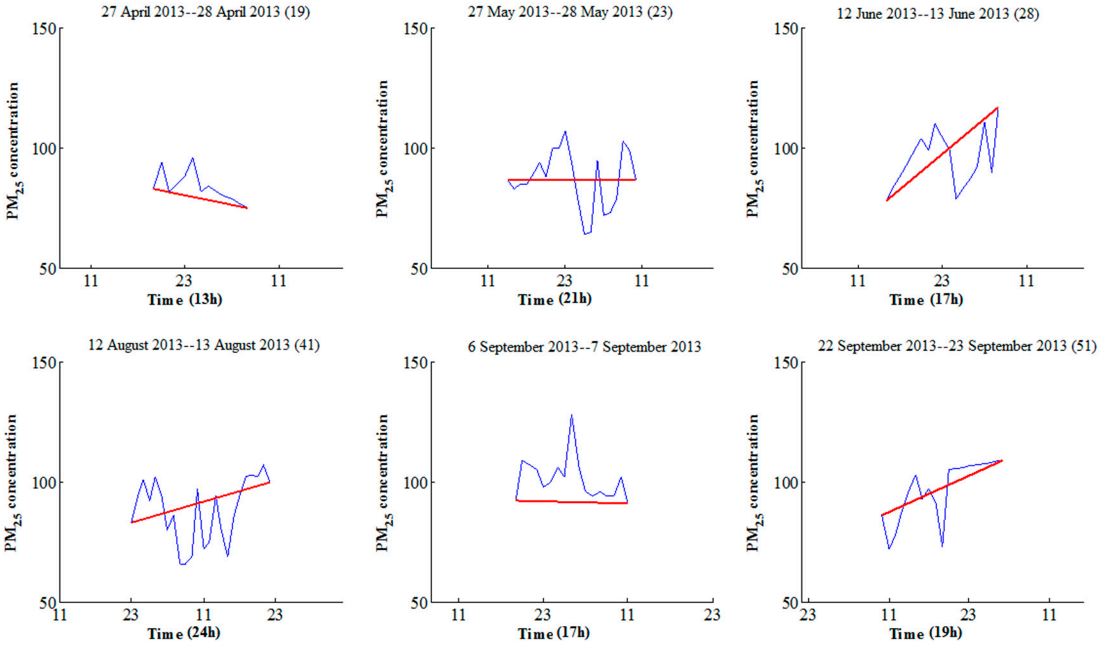

Figure S8. *Cont.*

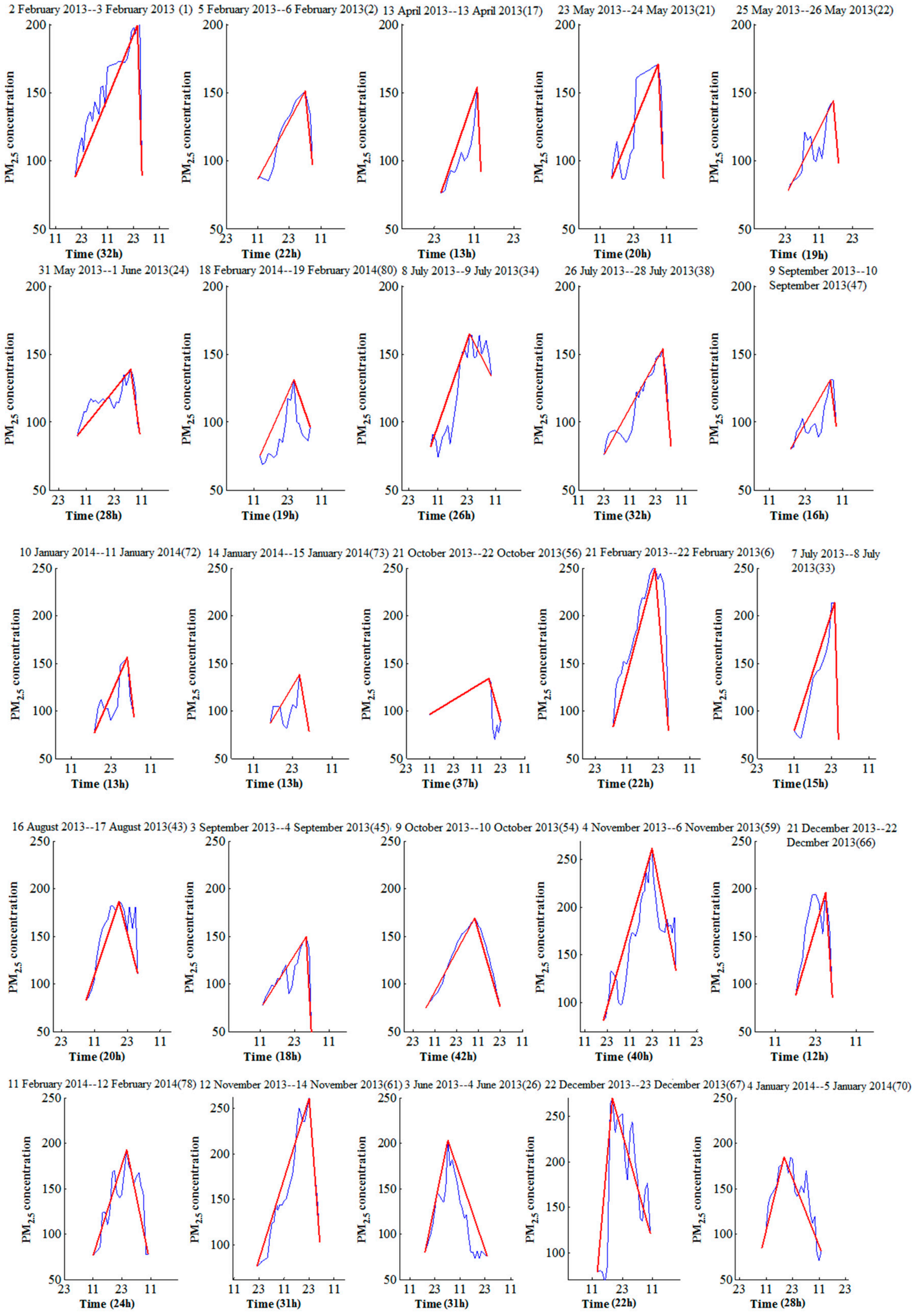

Figure S8. *Cont.*

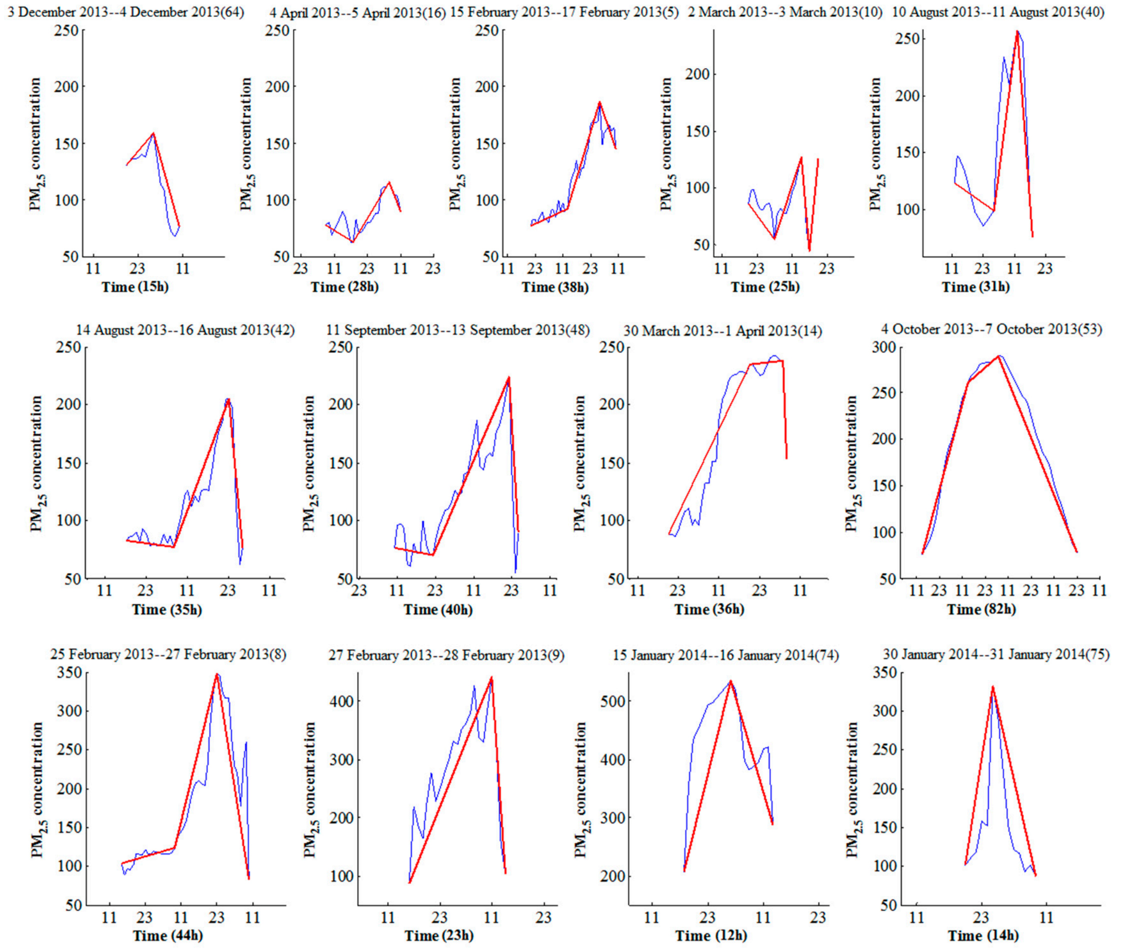

(b)

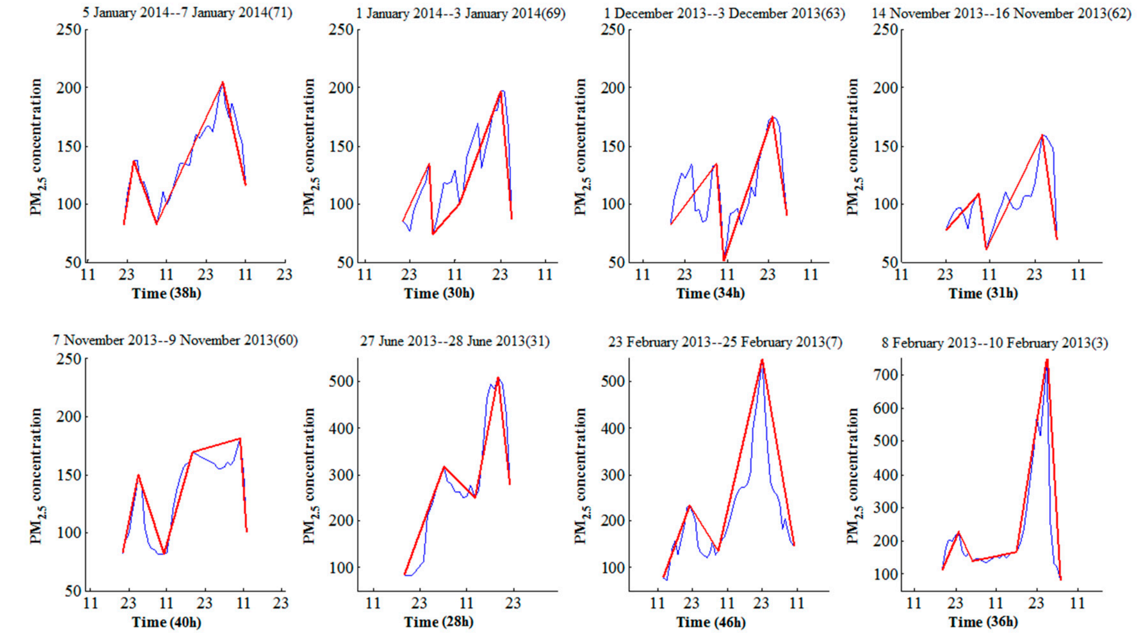

(c)

Figure S8. *Cont.*

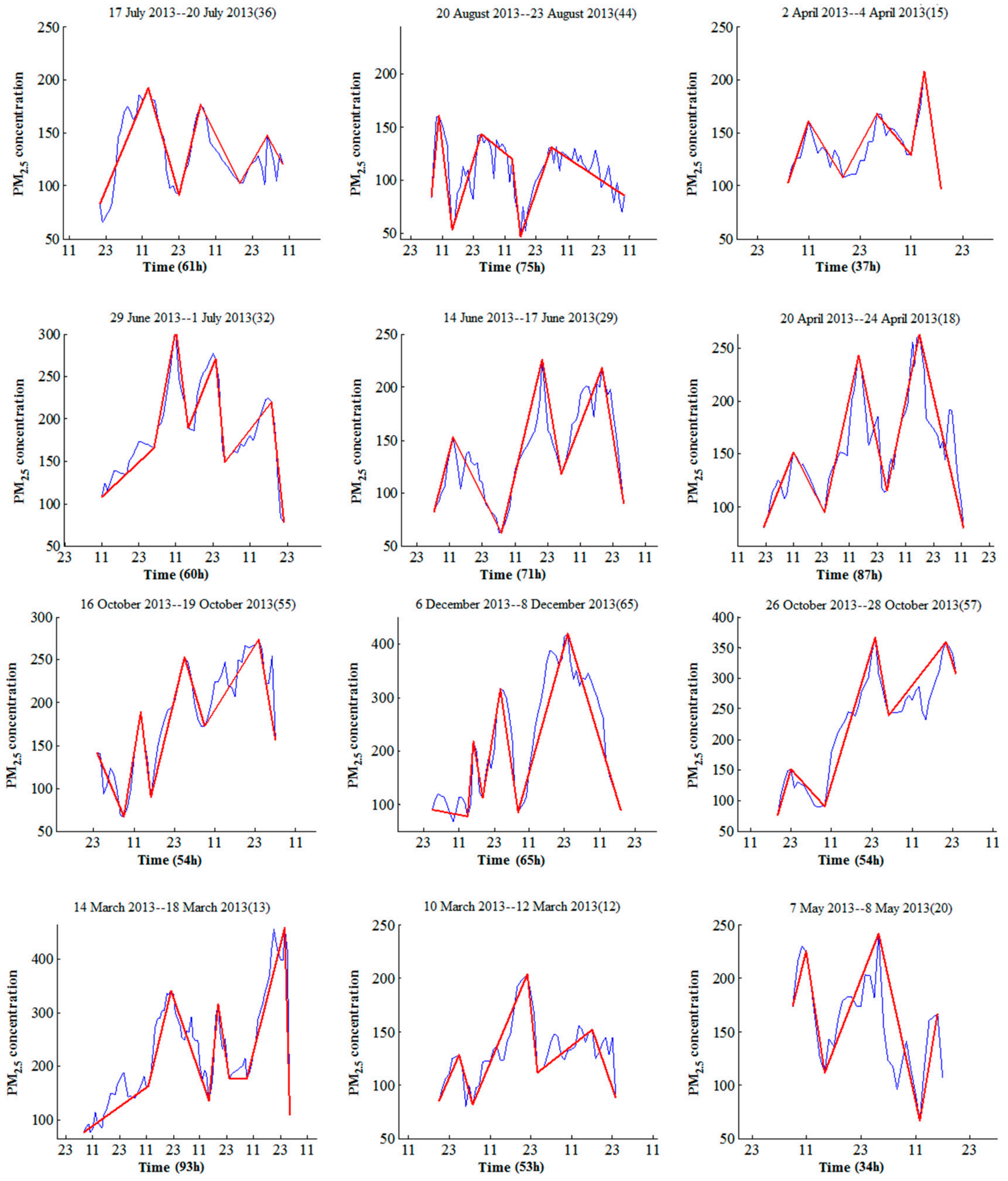

(d)

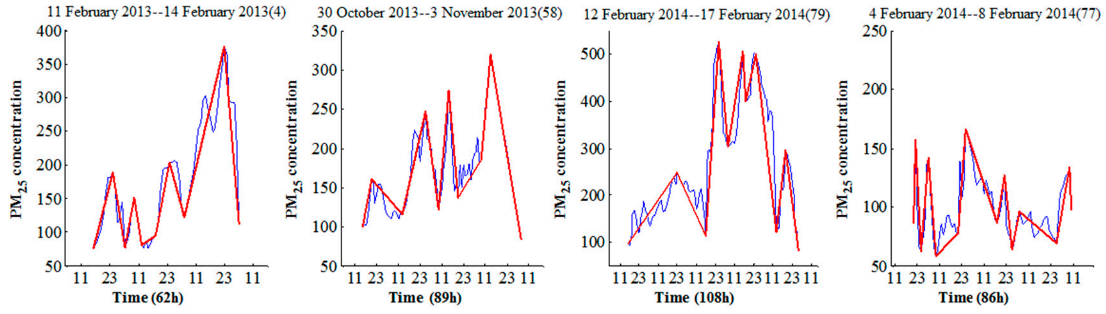

Figure S8. *Cont.*

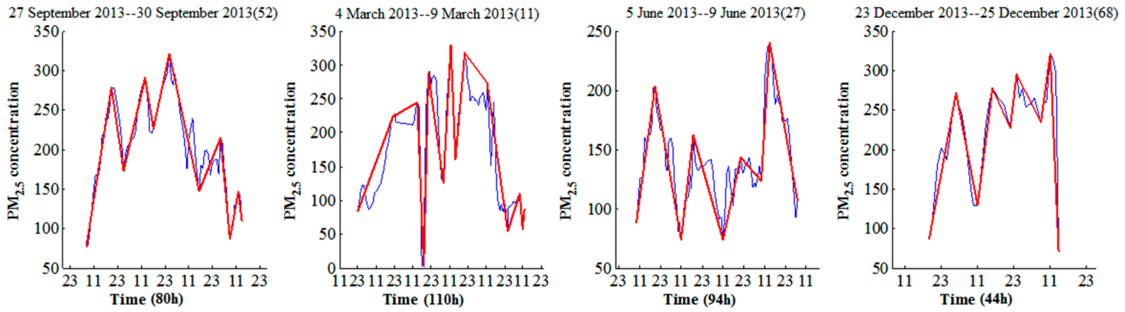

(e)

Figure S8. (**a**) $PM_{2.5}$ Pollution Episodes in first category of evolution mode; (**b**) $PM_{2.5}$ Pollution Episodes in second category of evolution mode; (**c**) $PM_{2.5}$ Pollution Episodes in third category of evolution mode; (**d**) $PM_{2.5}$ Pollution Episodes in fourth category of evolution mode; (**e**) $PM_{2.5}$ Pollution Episodes in fifth category of evolution mode.

Table S3a. Correlations between meteorological factors and $PM_{2.5}$ Pollution Episodes in the first category.

ID	C_ws	C_rh	C_blh	ws_ave	rh_ave	blh_ave
19	−0.06	−0.08	−0.42	2.47	0.55	385.11
23	0.37	0.19	0.03	2.15	0.71	596.83
28	−0.34	0.36	−0.58	2.33	0.64	773.11
41	0.30	−0.19	0.11	2.11	0.78	515.72
46	0.23	0.80	−0.89	1.67	0.78	487.47
51	−0.63	−0.18	−0.12	2.27	0.83	414.86

Table S3b. Correlations between meteorological factors and $PM_{2.5}$ Pollution Episodes in the second category.

ID	Season	C_ws	C_rh	C_blh	ws_ave	rho_ave	blh_ave	Cluster
21	Spring	−0.77	0.93	−0.84	3.90	0.48	849.94	C1
16	Spring	−0.64	0.87	−0.77	1.73	0.75	560.92	C1
38	Summer	−0.48	0.51	−0.07	1.62	0.90	265.07	C1
24	Spring	0.20	0.46	−0.30	3.50	0.40	914.45	C1
53	Autumn	−0.31	0.42	−0.02	2.61	0.66	426.93	C1
17	Spring	−0.57	0.40	−0.44	4.02	0.45	634.12	C1
34	Summer	−0.21	0.32	−0.70	1.87	0.85	493.17	C1
61	Autumn	−0.02	0.28	0.11	2.98	0.63	163.13	C1
54	Autumn	0.08	0.16	−0.07	3.64	0.65	666.93	C1
45	Autumn	0.81	0.00	−0.12	1.53	0.72	620.61	C2
42	Summer	0.39	−0.03	0.11	2.72	0.82	442.38	C2
33	Summer	0.19	−0.15	−0.14	3.85	0.64	1056.60	C2
26	Summer	0.19	−0.16	−0.36	2.85	0.54	714.20	C2
14	Spring	−0.07	−0.25	0.48	2.56	0.78	353.09	C2
56	Autumn	0.69	−0.41	0.32	2.63	0.62	483.46	C2
6	Winter	0.43	−0.43	0.31	2.33	0.31	723.97	C2

Table S3b. *Cont.*

ID	Season	C_ws	C_rh	C_blh	ws_ave	rho_ave	blh_ave	Cluster
48	Autumn	0.61	−0.44	0.54	3.30	0.71	580.09	C2
40	Summer	0.22	−0.53	0.45	2.85	0.77	626.69	C2
22	Spring	0.11	−0.55	0.59	3.19	0.70	650.62	C2
43	Summer	0.12	−0.60	0.30	2.27	0.80	467.86	C2
10	Spring	0.64	−0.71	0.78	2.84	0.37	538.23	C2
1	Winter	−0.73	0.91	−0.27	3.07	0.74	324.78	C3
2	Winter	−0.30	0.57	−0.51	1.97	0.65	333.38	C3
5	Winter	−0.59	0.70	−0.54	2.49	0.68	410.28	C3
64	Winter	−0.98	0.86	−0.74	2.06	0.52	81.42	C3
66	Winter	−0.66	−0.13	−0.81	0.91	0.46	22.11	C3
73	Winter	−0.26	0.17	−0.51	2.25	0.36	260.70	C3
72	Winter	0.08	−0.26	−0.21	2.19	0.30	49.43	C3
75	Winter	0.60	−0.11	−0.37	1.59	0.43	183.09	C3
8	Winter	0.41	−0.05	−0.29	2.44	0.58	424.88	C3
74	Winter	−0.60	0.53	−0.66	1.78	0.43	289.76	C3
67	Winter	0.15	0.76	−0.83	2.00	0.44	222.11	C3
70	Winter	0.19	0.58	−0.56	2.19	0.59	398.26	C3
59	Autumn	−0.49	0.73	−0.53	2.90	0.63	395.93	C3
47	Autumn	−0.83	0.92	−0.78	2.72	0.74	413.03	C3
9	Winter	−0.25	0.83	−0.62	3.92	0.55	559.04	C3

Table S3c. Correlations between meteorological factors and $PM_{2.5}$ Pollution Episodes in the third category.

ID	Season	C_ws	C_rh	C_blh	ws_ave	rho_ave	blh_ave	Class
35	Summer	−0.69	0.67	−0.59	1.95	0.78	675.93	C1
60	Autumn	−0.23	0.43	−0.50	2.68	0.52	217.11	C1
71	Winter	−0.59	0.57	−0.44	2.46	0.69	245.60	C1
63	Winter	−0.13	0.40	−0.25	2.06	0.47	87.04	C1
62	Autumn	−0.34	0.22	−0.12	2.58	0.46	218.03	C1
30	Summer	−0.10	−0.19	0.19	1.87	0.58	902.50	C1
39	Summer	−0.34	−0.08	−0.02	1.70	0.68	632.88	C1
37	Summer	−0.24	−0.14	−0.30	2.44	0.73	584.38	C1
50	Autumn	0.28	0.03	0.13	2.27	0.80	475.91	C1
49	Autumn	0.43	−0.65	0.48	3.02	0.79	558.67	C1
25	Summer	0.53	0.32	−0.09	3.30	0.55	964.17	C1
7	Winter	0.11	0.57	−0.37	1.95	0.44	451.56	C2
3	Winter	0.13	0.23	−0.38	2.40	0.59	366.79	C2
31	Summer	0.76	0.17	0.08	2.84	0.67	795.62	C2
69	Winter	0.12	−0.27	0.17	2.15	0.48	148.96	C2

Table S3d. Correlations between meteorological factors and $PM_{2.5}$ Pollution Episodes in the fourth category.

ID	Season	C_ws	C_rh	C_blh	ws_ave	rho_ave	blh_ave
65	Winter	−0.47	0.64	−0.51	1.63	0.61	234.17
20	Spring	−0.36	0.09	−0.34	3.49	0.38	1135.67
44	Summer	−0.35	0.36	−0.22	2.06	0.62	678.26
13	Spring	−0.13	−0.06	0.11	2.81	0.56	541.04
57	Autumn	−0.12	−0.15	0.18	1.76	0.66	217.66
29	Summer	−0.11	0.16	0.13	3.00	0.63	618.09
18	Spring	−0.10	0.22	−0.04	3.01	0.58	799.04
32	Summer	0.04	−0.26	0.11	3.26	0.74	720.90
36	Summer	0.07	0.56	−0.21	1.78	0.78	419.35
12	Spring	0.19	0.10	0.22	3.23	0.50	430.58
15	Spring	0.24	−0.01	0.26	3.49	0.40	592.75
55	Autumn	0.47	−0.11	−0.04	2.49	0.63	365.97

Table S3e. Correlations between meteorological factors and $PM_{2.5}$ Pollution Episodes in the fifth category.

ID	Season	C_ws	C_rh	C_blh	ws_ave	rho_ave	blh_ave
4	Winter	−0.39	−0.23	−0.23	2.73	0.41	531.88
11	Spring	−0.16	−0.15	−0.09	2.84	0.46	404.02
52	Autumn	−0.14	−0.13	−0.10	2.51	0.68	450.82
27	Summer	0.25	−0.27	0.14	2.20	0.82	526.51
58	Autumn	−0.32	−0.03	0.14	1.90	0.69	304.35
68	Winter	−0.28	0.51	−0.29	1.79	0.53	243.41

5. Illustrative Cases

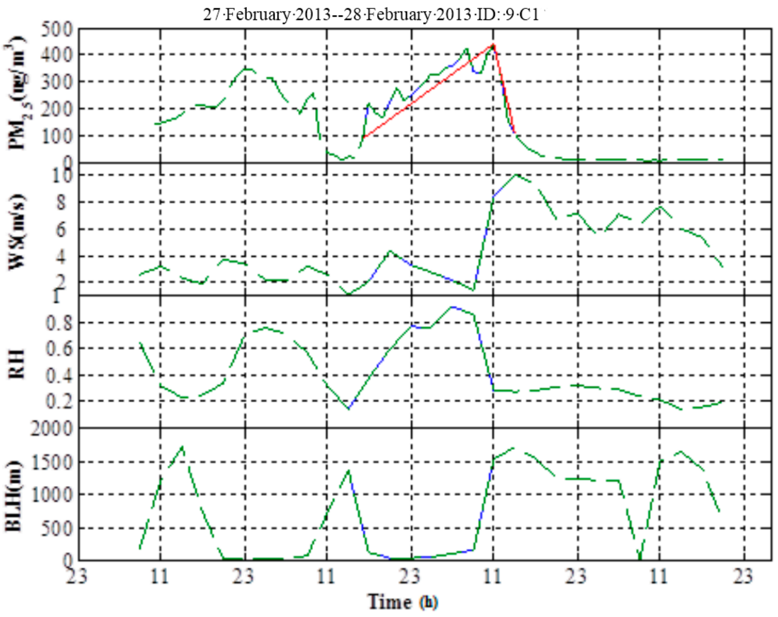

(a)

Figure S9. *Cont.*

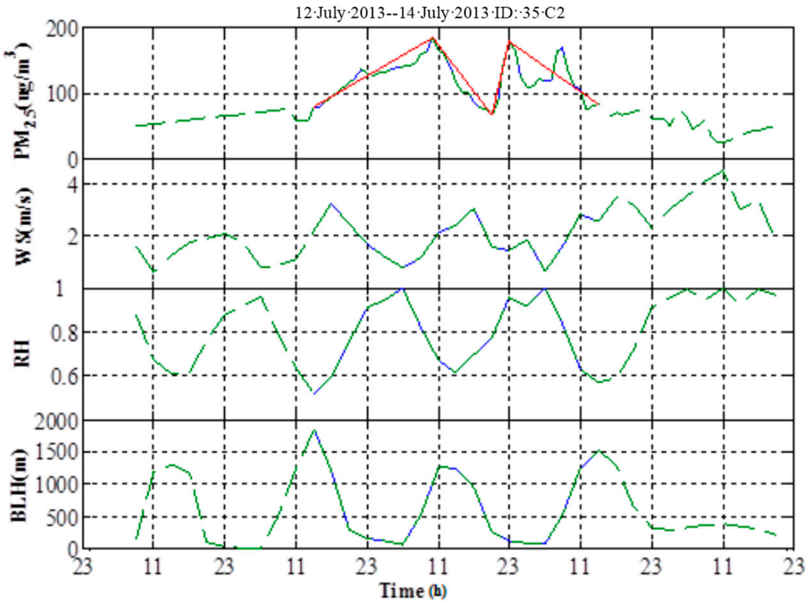

(b)

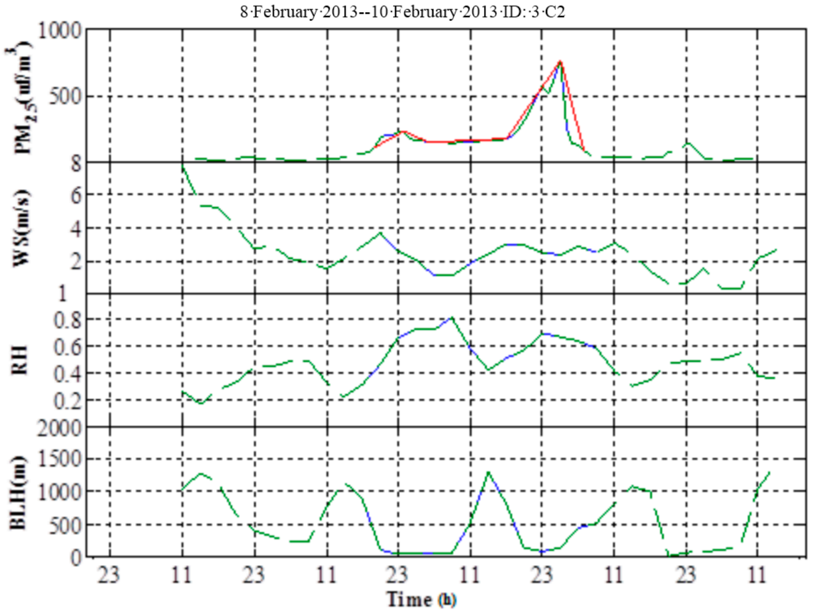

(c)

Figure S9. (**a**) Single peak, wind blowing-off; (**b**) Double peaks, synchronous variations; (**c**) Small-Big peak, multi-source emission.

© 2015 by the authors; licensee MDPI, Basel, Switzerland. This article is an open access article distributed under the terms and conditions of the Creative Commons Attribution license (http://creativecommons.org/licenses/by/4.0/).
